# Scheduling an appointment for MRI: patient perception of wait time and difficulty

**DOI:** 10.1186/s13584-025-00677-5

**Published:** 2025-03-26

**Authors:** Clara Singer, Noga Boldor, Sharona Vaknin, Liraz Olmer, Rachel Wilf-Miron, Vicki Myers

**Affiliations:** 1 The Gertner Institute for Epidemiology and Health Policy Research, Sheba Medical Center, 5266202 Tel Hashomer, Ramat-Gan, Israel; 2 School of Public Health, Faculty of Medical and Health Sciences, Tel Aviv University, Ramat Aviv, Israel

**Keywords:** Magnetic resonance imaging (MRI), Wait time, Patient perspective, Patient survey, Perceived difficulty

## Abstract

**Background:**

Wait times (WTs) for Magnetic Resonance Imaging (MRI) are rising in many countries. Long WT delay diagnosis and treatment, and affect patient satisfaction. Little research has examined the patient experience of scheduling and waiting for an MRI. This study aimed to assess difficulty of scheduling an appointment for MRI from patients’ perspectives; and to identify factors associated with longer WT and greater difficulty.

**Methods:**

An online survey of patients’ experience of scheduling an MRI was conducted in January–February 2023 among a representative sample of 557 Israeli adults. All participants had undergone an MRI in the public health system within the past year.

**Results:**

Median WT was 1–2 months and did not differ significantly by demographic variables or exam type. 28% considered the WT unreasonable. WTs ≥ 1 month were reported by two thirds of respondents; longer WT were reported for respondents who tried to get an earlier appointment compared to those who did not (*p* < 0.001). WT for radiology report was significantly related to shift (shorter WT for morning MRI exams, *p* = 0.045), sex (men reported shorter WT, *p* = 0.042) and age (over 55s reported shortest WT, *p* = 0.006). In a stepwise logistic regression modeling the probability of finding the process difficult, significant factors included time between referral and calling to schedule, tried multiple sites, tried to get an earlier appointment, WT for report, and overall reasonableness of WT.

**Conclusions:**

Many patients experienced some difficulty scheduling an MRI, particularly when calling multiple sites, since there is no centralized point of contact. HMO agreements can also lengthen the wait. Alongside objective metrics documented by service providers, it is important to consider patients’ perspectives in scheduling imaging. While efforts have been made in recent years to tackle MRI WT, adding scanners and personnel, the majority of patients wait at least a month, and the process of scheduling and waiting for an appointment can be challenging. Policy changes, including greater transparency of WTs in different institutions, and a centralized booking center for MRI, could be considered to streamline the appointment process and reduce the challenges patients face.

## Introduction

Magnetic resonance imaging (MRI) has become an indispensable feature of the diagnostic landscape. Demand for this high-cost imaging resource has dramatically increased over the last decades, driven by the evolving clinical use for the modality [1, 2], as well as by patients' expectations to shift to this novel, radiation-free examination. The effect of patient expectation is expressed in the figure that more than one third of US physicians would order an MRI for back pain if the patient requested this exam, even if they think that it is unnecessary [3].

Alongside increasing demand, wait times for MRI are rising in many countries, and insufficient access has been described [4]. The COVID-19 pandemic increased wait times by creating backlogs, with 21% of citizens of the European reporting that they missed a medical examination or treatment during the pandemic [5]. In Israel, a 47.5% reduction in MRI utilization was evident during the first peak of the pandemic wave, compared to the parallel period in the previous year [6]. Long wait times both delay diagnosis, and consequently treatment, and affect patient satisfaction [7–9]. Furthermore, the long WT for MRI can lead clinicians to refer for other tests while waiting, which may not be sufficient, and many patients end up getting an MRI, thereby leading to unnecessary irradiation and increased costs for the health service [10].

In response to patient dissatisfaction with long wait times for MRI in Israel, a reform was launched in 2015. The national program to shorten MRI WTs included addition of scanners, and personnel, round-the-clock testing, training programs and financial incentives for providers [11]. Eight years following the reform, WTs are still long. Israel has a relatively low number of MRI scanners per population compared to the OECD average, though the number of exams is ranked higher. In 2015 there were 4 scanners per million population in Israel, compared to 15.8 OECD average [12]. In 2021, Israel was rated in the bottom 5 countries for scanners per population, with just 5/million while the OECD average was 18/million [13]. Equally in 2021 Israel conducted 47 MRI exams/1000 population, comparable to 51/1000 for the UK, and 57/1000 for Australia, but lagging behind the OECD average of 84/1000. This suggests that the Israeli health system makes efficient use of its available scanners.

In Israel, the mandatory National Health Insurance Law provides coverage to all citizens through four competing HMOs, and is financed from residents’ taxes contributed to Social Security (State Health Insurance Law, 1994). Data are regularly collected from providers on WTs for MRI. Surveys are a widely used means of obtaining patients’ perspective on health services and can often provide a good indication of WT [14]. However patients’ perspectives on the imaging pathway are rarely evaluated. Previous patient satisfaction surveys in imaging have focused mostly on waiting on the day of the appointment and of the physical conditions of the test itself [8, 15–17]. Research on wait times for an appointment are less common have been largely restricted to waiting for surgery, or some patient surveys which examined satisfaction related to wait time for appointments in primary or specialist care [18]. Research on wait times for imaging appointments is sparse, and often relies on system data [19].

The current study used a survey to obtain patients’ perspectives on the process of scheduling and waiting for an MRI appointment in the public health system, as part of a broader process of evaluating the MRI process in Israel. Beyond waiting times, the survey aimed to evaluate how easy or difficult patients found the process, and what factors are related to finding the process difficult.

## Aims

The aims of this research were.

1. To assess reported WTs for MRI appointment and for MRI results from the patients’ perspectives and how reasonable they consider those times;
2. To assess the perceived difficulty of scheduling an appointment for MRI and which aspects patients find the most difficult;
3. To identify factors associated with longer WT for appointment and for radiology report, and with greater perceived difficulty.

## Methods

### Data collection

A survey was developed based on previous qualitative research, which included patient focus groups, in diverse patient populations and geographical regions, which helped elucidate the most important themes and issues related to the process of scheduling an MRI exam in the public healthcare system. The focus groups showed a lot of frustration with the process of scheduling an MRI appointment, as well as variation in waiting times, with some people waiting for months, while others found the process less problematic. This led us to ask which patients perceive the process to be more difficult and which factors are related to greater difficulty, as well as to longer WTs.

The survey was distributed via an internet panel survey in February 2023 to 26,596 people. Of those, 3876 entered the survey, and 500 who were eligible, having undergone MRI in the public health system in the past year, completed the survey. Purposive sampling was conducted to get a sample representative of the 4 HMOs, and 4 geographic regions. Since there was not adequate representation of all HMOs and geographic regions, the survey was sent out a second time to 25,091 people, of which 4815 clicked on the survey, and 57 people who fit the inclusion criteria (having had an MRI in the past year, and belonged to the relevant under-sampled groups) completed the survey.

Non-responders (those who did not complete the survey) were slightly younger than responders (mean age 38.8 ± 14.2 years vs. 44.2 ± 14.5 years)(*p* < 0.001). There was also a larger proportion of women among non-responders (61.7%) compared to responders (56.6%).

The final survey sample included 557 people age 18 + who had undergone an MRI in the last 12 months (January 2022-February 2023) in the public health system.

There were three screening questions, which assessed: 1. If the person had undergone an MRI, 2. If the MRI was reimbursed by the HMO, and 3. When the MRI occurred. All those who fit the inclusion criteria of publicly reimbursed MRI within the last 12 months entered the survey. Respondents were asked how long they waited for an appointment, and for the test results (radiology report), and about the difficulty of scheduling an appointment. Demographic data were collected on age, sex, education, income, religion, region of residence and HMO where they are insured. In addition, different appointment characteristics were collected such as type of MRI performed, existence of prior imaging, morning or evening shift when the exam was performed, if they tried to get an earlier appointment, and if they tried multiple sites in order to get an appointment.

Wait time for appointment (WTA) was measured in categories to facilitate recall: “how much time passed from the moment you tried to set an appointment until the day of the MRI?" Up to 2 weeks, 2–4 weeks, 1–2 months, 2–4 months, 4–6 months, over 6 months. Wait time for an appointment was categorized as a binary variable for some analyses as up to or over 1 month.

Wait time for report (WTR) was also measured in categories: "how long did it take to get the results?" Up to 1 week, 1–2 weeks, 2–4 weeks, over 1 month, did not receive. Wait time for results was categorized as a binary variable for some analyses as up to or over 2 weeks.

Difficulty and reasonableness of the process were measured on a scale of 1–5. Overall difficulty was categorized as a binary variable for some analyses as No difficulty/Not so difficult (score 1–2) or So So/Quite/Very difficult (score 3–5).

Finally, the respondents reported their place of residence, and the institution and location where they underwent the MRI examination. The distance of the exam from the place of residence is calculated and classified into categories: in the same city (matched city of residence and city of exam), 3–10 km, 10–20 km, 21–30 km, 31–50 km, and over 50 km.

### Data analysis

Descriptive statistics were used to analyze the characteristics of the sample.

Since disparities exist in other areas of health, we wanted to examine whether there are disparities in waiting time for an MRI, and therefore looked at the association between WT and demographic factors, as well as clinical and system factors which might be related, like HMO, type of scan, and time of shift, to see if these are related to WT. We also wanted to assess which factors are related to perceived difficulty of getting an appointment, whether it is a function of WT or related to other factors which are part of the scheduling process.

We performed univariate analyses to explore the association between wait time for appointment (WTA), wait time for report (WTR) and overall difficulty and other variables: prior imaging (yes/no), shift during which the MRI scan was performed (morning, afternoon, evening, night), MRI organ (abdomen/pelvis, neurological, breast, skeleton), tried to get earlier appointment (yes/no), HMO (1–4), sex, age group (18–34, 35–54, 55 +), religion (Jewish, other), self-reported income (below average, average, above average), education (high school, further education beyond high school, university), geographic region (North/Haifa, Center, Jerusalem, South), WT for HMO approval, i.e. financing of the exam (up to 1 week, 1–2 weeks, 2–4 weeks, > 1 month), reasonableness of WT (reasonable, somewhat reasonable, in the middle, somewhat unreasonable, very unreasonable). For subgroup analyses, binary variables were used for WT and difficulty. Categorical variables are reported as percentages and were compared with Pearson's chi-squared test or Fisher's exact test when the value of any expected cell was less than five.

Bonferroni correction was applied to adjust for multiple comparisons in univariate analyses, thus using a more stringent *p* value according to the number of variables tested (alpha divided by the number of tests performed).

We also analyzed WTA, WTR and overall difficulty as ordinal variables. For this purpose, we looked for association with gender, HMO, geographic region, religion, MRI shift and MRI organ using Wilcoxon Two-Sample Test or Kruskal–Wallis test.

Based on the results of the univariate analysis we proceeded with multivariate models only for the overall difficulty variable. To capture the contribution of each factor we constructed stepwise logistic regression models. The probability of the overall process being difficult (score between 3 and 5; where 1- not difficult, 2- not so difficult, 3- so so, 4- quite difficult, 5- very difficult) was modeled. Sex, HMO and age group were forced into the model and therefore were included as covariates.

Statistical analysis was performed using SAS Enterprise Guide v.8.3. A 2-tailed P-value of less than 0.05 was considered significant.

## Results

The survey sample included 557 respondents. The sample description is presented in Table 1. All HMOs and geographic regions were represented, as well as Jewish and Arab populations, although the Arab population, comprising 20% of the general population, was underrepresented. Men were also slightly under-represented, as was the smallest HMO. More than half of respondents had university education and about a third reported below average income.

**Table 1:** Sample description including patient characteristics and appointment characteristics, N = 557

Variable		N	%
Patient characteristics			
Sex (Missing = 9)	Female	315	56.6
	Male	233	41.8
Age	18–34	185	33.2
	35–54	248	44.5
	55 +	124	22.3
Religion (Missing = 1)	Jewish	514	92.3
	Muslim, Druze, Christian	42	7.6
Region	North/Haifa	160	28.7
	Centre	281	50.4
	Jerusalem	58	10.4
	South	58	10.4
Education	Up to high school	113	20.3
	Further education beyond high school	130	23.3
	University	314	56.4
Self-reported Income (Missing = 45)	Below average	189	33.9
	Average	131	23.5
	Above average	192	34.5
HMO (Missing = 7)	1	250	44.8
	2	166	29.8
	3	78	14
	4	56	10
Appointment characteristics			
Type of MRI*	Abdomen/pelvis	89	17.4
	Neurological	276	54.04
	Breast	27	5.34
	Skeleton	119	23.34
Prior imaging	Yes	334	60
	No	223	40
MRI shift	Morning	151	27.1
	Afternoon	141	25.3
	Evening	124	22.3
	Night	141	25.3
Tried to get an earlier appointment	Yes	283	50.8
	No	274	49.2
No. of sites tried to schedule	1 site	349	62.7
	2 sites	144	25.9
	> 2 sites	64	11.5

^*^We dropped observations with two different types of MRI, and those with type "other", 511 observations left

The most commonly reported MRI was neurological (head/brain) (54%), which is the most frequent exam (as reported by suppliers). MRI shift was almost equally distributed, with slightly more during the morning shift (*n* = 151, 27.1%). Sixty percent of respondents, underwent additional imaging procedures before their MRI scan. Among those who received preliminary imaging tests, computed tomography (CT) scans were the most common, with 43% of participants undergoing this imaging modality (data not shown). About half of the respondents tried to get an earlier appointment and 37% called more than one site to try to get an appointment. When asked how reasonable they considered the WT, 45% considered it reasonable or very reasonable, 28% considered the WT unreasonable or very unreasonable, and 27% rated intermediate.

Respondents were asked to rate on a scale of 1–5 the difficulty they experienced at different stages of the process. The highest difficulty was rated for the stage of scheduling the appointment itself (median 3), while a median difficulty rating of 2 was found for getting the referral, getting the test approved by the HMO (reimbursement form), and knowing where to call. Thirty percent rated scheduling an appointment as quite or very difficult, compared to 18% for getting the referral, and 23% for getting the results.

**Fig. 1:**
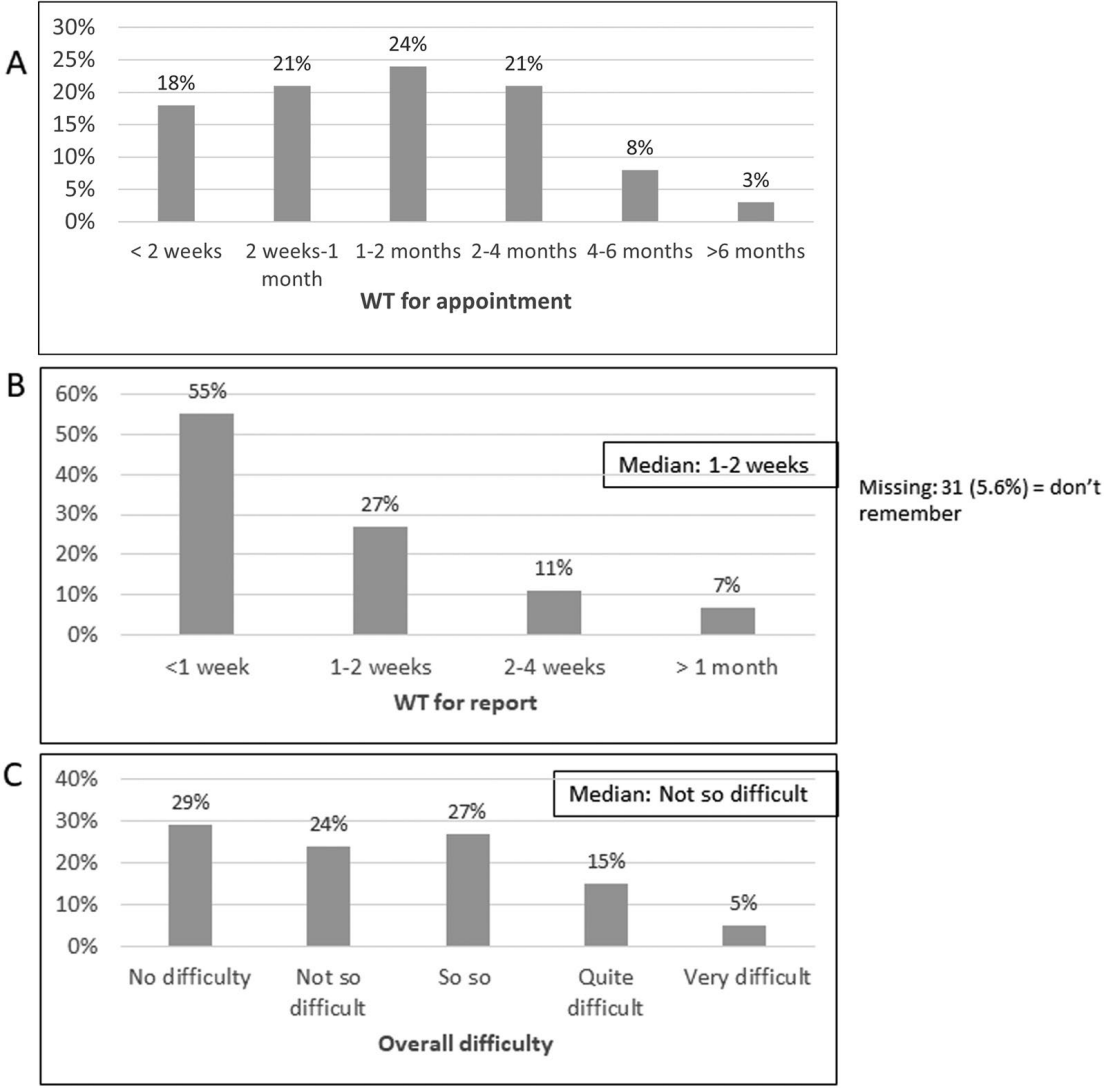
Distributions and medians of the three dependent variables: **a** WT for appointment, **b** WT for report, and **c** Overall difficulty

Figure 1 shows the distribution of the three dependent variables we further explored, all three being ordinal variables: "WTA" (1: < 2 weeks, 2: 2 weeks-1 month, 3: 1–2 months, 4: 2–4 months, 5: 4–6 months, 6: > 6 months), "WTR" (1: < 1 week, 2: 1–2 weeks, 3: 2 weeks-1 month, 4: > 1 month, 5: did not receive) and "overall difficulty" (1: no difficulty, 2: not so difficult, 3: So so, 4: quite difficult, 5: very difficult). Median WT for appointment was 1–2 months. Moreover, around a third of respondents reported WTA over 2 months. WT reported by patients was similar to WT reported by suppliers as recorded in the Ministry of Health's computer systems. Median WTR was 1–2 weeks. Median overall difficulty was the category "2: not so difficult".

**Fig. 2:**
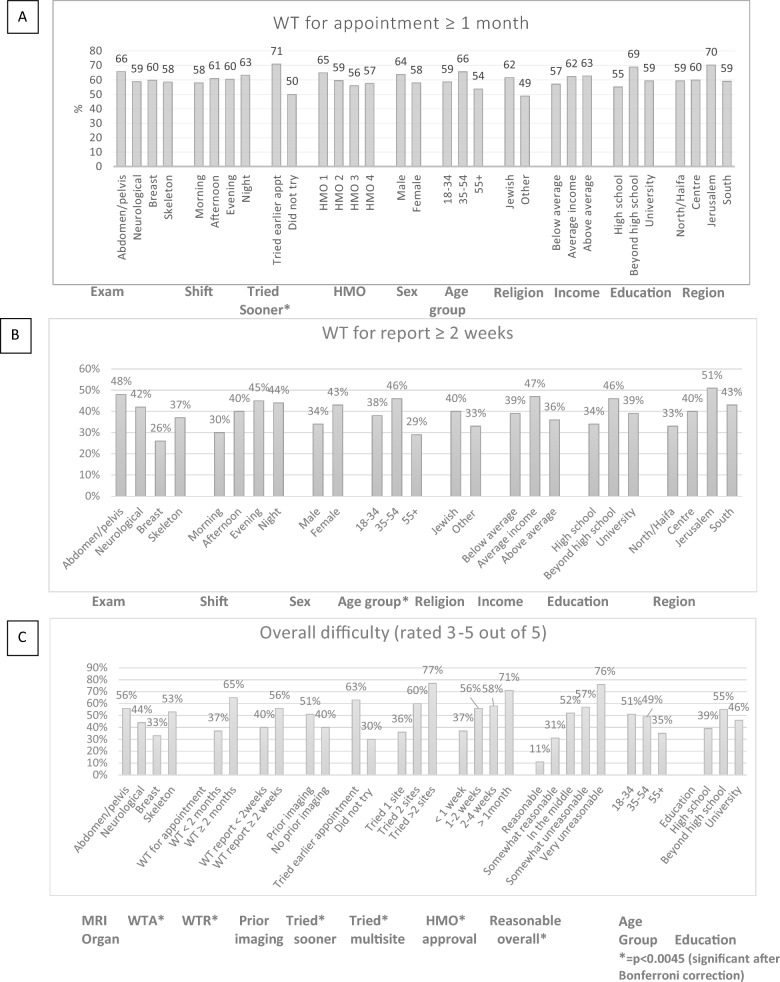
Chi-square associations between three dependent variables and clinical/demographic variables **a** WT for appointment, **b** WT for report, **c** Overall difficulty

When examining WTA as a binary variable (WT < 1 month, WT ≥ 1 month), there was no significant difference according to prior imaging, shift, exam type, HMO, or demographic variables including age, sex, education, income, region or religion (see Fig. 2A). Longer WT were found across respondents who tried to get an earlier appointment compared to those who did not (*p* < 0.001) (remained significant after Bonferroni correction: alpha cutoff = 0.0045).

When examining WTR as a binary variable (< or ≥ 2 weeks), it was significantly related to shift (shorter WT for morning MRI exams, *p* = 0.045)(NS after multiple comparisons), sex (men reported shorter WT, p = 0.042) (NS after multiple comparisons) and age (over 55s reported shortest WT, *p* = 0.006) (see Fig. 2B). WTR did not differ by exam type, HMO, region, religion, education or income.

When examining overall difficulty as a binary variable (score 3–5 out of 5) significant associations (after Bonferroni correction for multiple comparisons) were found between degree of difficulty and WTA (p < 0.0001), WTR (*p* < 0.001), trying to get an earlier appointment (*p* < 0.0001), calling multiple sites to get an appointment (*p* < 0.0001), WT for HMO approval (*p* < 0.001), and WT reasonableness (*p* < 0.001), (see Fig. 2C). More difficulty was reported by respondents with longer reported WT for appointment, longer reported WT for results, those who tried to get an earlier appointment, those that tried at multiple sites to schedule an appointment, respondents with longer reported WT for HMO approval and those who perceived the WT as very unreasonable. Also, younger people and people with education beyond high school perceived the process as more difficult than over 55s or those with high school or university education, respectively, though these did not remain significant after multiple comparisons.

When examining WTA, WTR and Overall difficulty as ordinal variables, the only association that came out significant was between WTR and MRI shift (shorter WT for morning shift, *p* = 0.0157) (Table 2). However this was no longer significant after Bonferroni correction given we did six tests (0.05/6 = 0.0083).

**Table 2:** Analysis of Waiting time for appointment, Waiting time for report and Overall difficulty as function of different explanatory variables. Wilcoxon Two-Sample Test/Kruskal–Wallis test, N = 557

	Waiting time for appointment^1^			Waiting time for report^2^			Overall difficulty^3^		
Variable	N	Median (Q1,Q3)	P-value	N	Median (Q1,Q3)	P-value	N	Median (Q1,Q3)	P-value
Gender:			0.4698			0.0577			0.5071
Male	228	3 (2,4)		222	2 (1,3)		233	2 (1,3)	
Female	304	3 (2,4)		295	2 (2,3)		315	2 (1,3)	
HMO:			0.7055			0.337			0.9647
1	245	3 (2,4)		233	2 (1,3)		250	2 (1,3)	
2	160	3 (2,4)		155	2 (2,3)		166	2 (1,3)	
3	75	3 (2,4)		78	2 (2,3)		78	2 (1,3)	
4	54	3 (2,4)		53	2 (2,3)		56	2 (1,3)	
Region:			0.1439			0.1697			0.4613
Haifa and North	157	3 (2,4)		151	2 (1,3)		160	2 (1,3)	
Central area	271	3 (2,4)		266	2 (1,3)		281	2 (1,3)	
Jerusalem area	57	3 (2,4)		55	3 (2,3)		58	3 (2,3)	
Beer-sheva and South	56	3 (2,4)		54	2 (2,3)		58	3 (2,3)	
Religion:			0.097			0.7936			0.1247
Jewish	499	3 (2,4)		487	2 (1,3)		514	2 (1,3)	
Other	41	2 (2,3)		39	2 (2,3)		42	2 (1,3)	
MRI shift:			0.4282			*0.0157			0.1393
Morning	145	3 (2,4)		143	2 (1,3)		151	2 (1,3)	
Afternoon	138	3 (2,4)		134	2 (2,3)		141	3 (2,3)	
Evening	121	3 (2,4)		118	2 (2,3)		124	2 (1,3)	
Night	137	3 (2,4)		131	2 (2,3)		141	3 (1,3)	
MRI organ:			0.2329			0.1149			0.4396
Abdomen/pelvis	86	3 (2,4)		86	2 (2,3)		89	3 (1,3)	
Neurological	267	3 (2,4)		258	2 (2,3)		276	2 (1,3)	
Breast	27	3 (1,5)		27	2 (1,3)		27	2 (1,3)	
Skeleton	116	3 (1,4)		112	2 (1,3)		119	3 (1,3)	

*Statistically significant at the level of P ≤ 0.05

^1^1- < 2 weeks, 2- 2 weeks-1 month, 3- 1–2 months,4- 2–4 months, 5- 4–6 months, 6- > 6 months

^2^1- < 1 week, 2- 1–2 weeks, 3- 2 weeks-1 month, 4- > 1 month, 5- did not receive

^3^1- not difficulty, 2- not so difficult, 3- So so, 4- quite difficult, 5- very difficult

When building a stepwise logistic regression for modeling the probability of the overall process being difficult (score between 3 and 5; where 1- not difficulty, 2- not so difficult, 3- So so, 4- quite difficult, 5- very difficult), five explanatory variables were significant and selected into the model: "WT from referral to call" (*p* = 0.0005), "WT time for report" (*p* = 0.032), "tried earlier appointment" (*p* < 0.0001), "tried multiple sites" (*p* < 0.0001) and "reasonable overall" (*p* < 0.0001) (Table 3). Sex, HMO and age group were forced into the model and none were significant. Thus, patients who tried to reschedule and get an earlier appointment are 2.482 times more likely to report overall difficulty as compared to those who did not (95% CI 1.596–3.86) and those who tried at multiple sites were almost twice as likely (OR 1.922, 95% CI 1.397–2.644) to report difficulty. In addition, patients who waited longer from the referral until they called to get an appointment, patients who waited longer for the report results, patients who tried to get an appointment in more than one site and patients who found the WT more unreasonable were more likely to report overall difficulty. The Area Under the Curve for the selected model was 0.802.

**Table 3:** Stepwise logistic regression modelling the probability of the overall process being difficult (score between 3 and 5), N = 550

	Odds Ratio Estimates			Pr > ChiSq
	Point Estimate	95% Wald Confidence Limits		
Intercept				< .0001^*^
Sex, Female vs Male	0.969	0.632	1.484	0.884
HMO, vs 4				0.5395
1	0.676	0.329	1.386	
2	0.85	0.403	1.794	
3	0.603	0.257	1.417	
Age group, vs 55 +				0.3404
18–34	1.532	0.85	2.761	
35–54	1.397	0.807	2.419	
WT referral to call^1^, 1- unit increment	1.309	1.125	1.523	0.0005^*^
WT for report^2^, 1- unit increment	1.265	1.02	1.568	0.032^*^
Tried earlier appointment, Yes vs No	2.482	1.596	3.86	< .0001^*^
Tried multiple sites^3^, 1- unit increment	1.922	1.397	2.644	< .0001^*^
Reasonable overall^4^, 1- unit increment	1.615	1.352	1.929	< .0001^*^

*Statistically significant at the level of P ≤ 0.05

^1^1- < 1 week, 2-1–2 weeks, 3-2 weeks–1 month, 4-1–2 months, 5- > 2 months

^2^1- < 1 week, 2-1–2 weeks, 3-2 weeks–1 month, 4- > 1 month, 5- did not receive

^3^1-1 site, 2- 2 sites, 3- more than 2 sites

^4^1- reasonable, 5- not reasonable

**Fig. 3:**
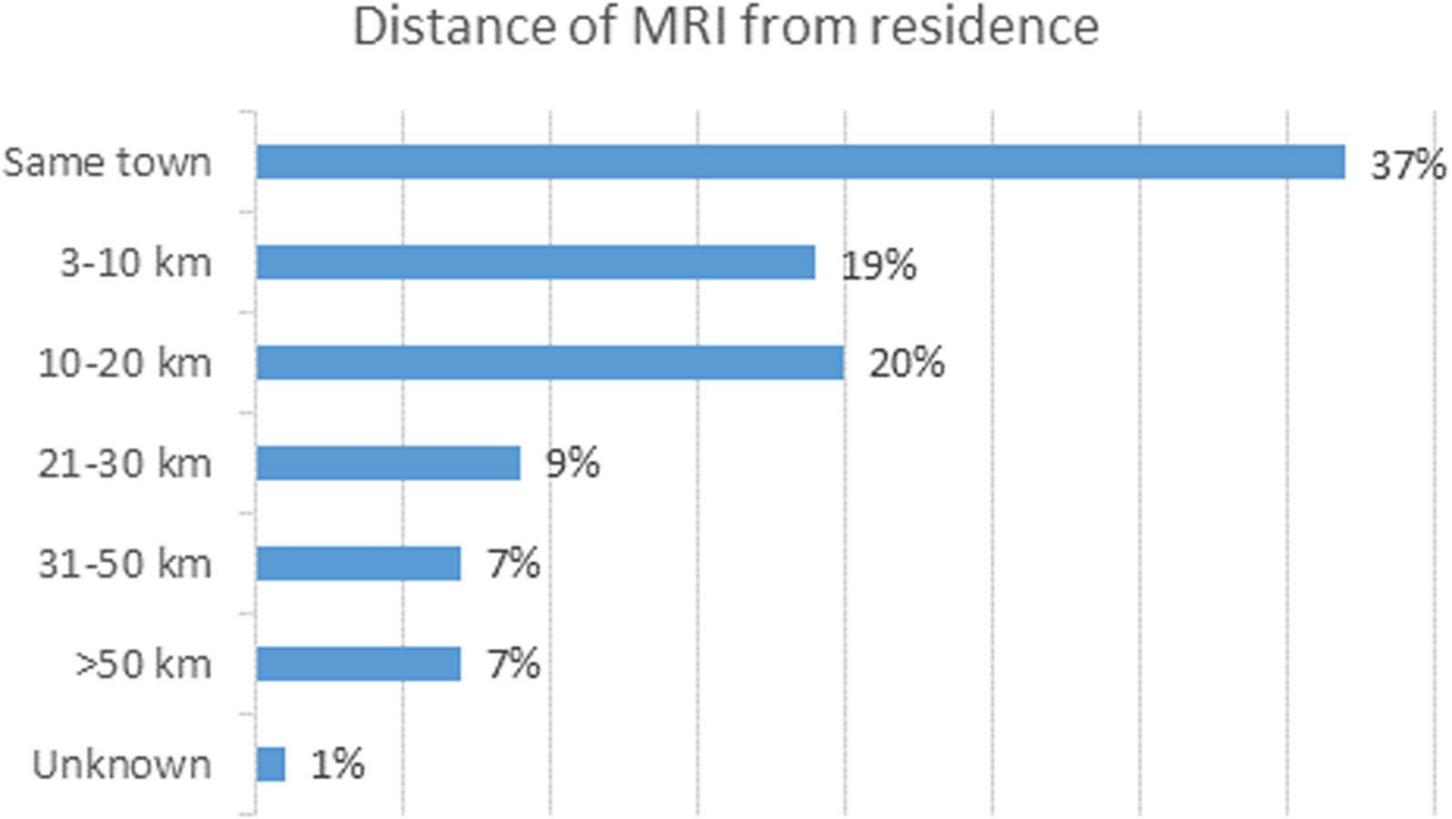
Distribution of distance of MRI exam from residence

Finally, Fig. 3 shows the distribution of the distance between patient residence and site where MRI was performed. Over a third of the respondents indicated that they underwent the MRI test in their town/city of residence, while, 14% travelled more than 30km for the test.

## Discussion

As demand for imaging—and consequently wait times for MRI—increase, patient satisfaction can be negatively affected. Since MRI is often part of the diagnostic process, each extra day of waiting can add markedly to the stress patients experience. This study—based in Israel but likely relevant to patients in other countries—examined the process of scheduling and waiting for an appointment from the patients’ perspective.

A patient survey was distributed among patients who underwent an MRI in the past year in the Israeli public healthcare system. Median WT was 1–2 months. While around a third of respondents reported waiting over 2 months for an MRI, and almost two thirds waited at least 1 month, about a third considered the WT for an MRI to be reasonable. These figures are comparable with a third of respondents who were satisfied with WT for a community-based specialist in a 2018 survey of healthcare in Israel [20]. To compare with MRI in other countries, in the UK WT for an MRI is estimated to be between 6 and 18 weeks, with a reported 20% waiting over 6 weeks [21]. Reported median WT in Canada was 12–20 weeks in 2022 [22], and in Norway 9–12 weeks in 2021, from self-report data [23].

Patient surveys are not routinely conducted regarding scheduling and WTs – however, the current survey showed that WT for MRI reported by patients was similar to WT reported by suppliers [11]. Despite the possibility of recall bias, this approximation strengthens the validity of self-reported WT. Another survey examining patient reports of WT for specialists in Israel also found correlation between survey and system data on WT. [14]

Israel’s public healthcare system is served by four competing healthcare maintenance organizations (HMOs), which patients choose, and can move between. All members have access to a statutory benefits package. Somewhat surprisingly, reported WT did not differ significantly between HMOs, geographical regions, exam type, or by demographics. This suggests uniform management across the country and across providers, with patients from different population groups reporting similar waiting times.

Many patients did report difficulty with the scheduling process. Perceived difficulty was associated with wait time and with multiple attempts to schedule an appointment. Patients may ‘shop around’ for the quickest appointment since WTs are often extensive for MRI. In Israel, HMOs have agreements with certain hospitals/providers which limit where patients can or can’t access imaging tests. This means for example that the 53% of the population insured by the largest HMO can usually only access tests in that HMO’s facilities. Scheduling an MRI involves calling the hospital and providing the appropriate paperwork for the test, including the referral and reimbursement form from the HMO. There is no central switchboard for scheduling imaging appointments. Moreover, waiting time for MRI at different hospitals are not published and accessible to the general public, therefore patients have to call each one to find out where the shortest WT are, and sometimes schedule multiple appointments. Several strategies have been proposed for streamlining imaging appointment scheduling including having an open schedule rather than fixed blocks, and reducing the complexity of the scheduling system for patients [4]. However, the specific characteristics of MRI and its high cost mandate that a radiologist will review each referral and decide on its appropriateness and the exact modality (i.e. with or without gadolinium injection). This dictates that patients are unable to self-schedule the exam, a fact that has been described as a barrier—potentially increasing cancellations and adding burden to administrative staff [24]. However, greater transparency of hospital WT could help patients to more easily schedule the closest appointments.

Another factor which may increase perceived difficulty in scheduling an MRI is that a large proportion of patients have already undergone previous imaging tests, with their associated difficulty and wait time, by the time they are referred for MRI. Thus, the effort to obtain an appointment and the additional wait for another appointment are cumulative. Some patients had already had more than one prior imaging test. While imaging tests are vital in diagnosis, it has been suggested that physicians may sometimes refer to a test with a shorter WT, in lieu of directly referring to MRI with known long queues, and that some patients may be undergoing redundant tests, as well as incurring additional costs to the health system. [10]

It is important to note that not all patients immediately call the hospital after receiving a referral from their doctor—around a third waited more than a month. This is important to take into consideration when measuring WT, which should not automatically be measured from the date the referral was issued. This delay could be due to multiple factors, including fear of diagnosis, seeking a second opinion, practical considerations, the problem improving/resolving etc. To take this delay into consideration, a meaningful measure of WT for MRI can be from the first date the patient contacted the provider to schedule an appointment until the appointment itself or report of results.

Most respondents reported receiving the results within two weeks but over a third waited longer or never received the report. Men reported shorter WT for results compared to women. Israeli hospitals generally tell patients the expected timeframe for results, which can be anything from 1 to 3 weeks—this helps to manage expectations. Many imaging departments are short-staffed for radiologists, which lengthens the turnaround for MRI reports.

Little research has examined the patient experience of scheduling appointments. A nationally representative patient survey which examined scheduling a specialist physician appointment in Israel found that younger and more educated patients were more likely to try to get an earlier appointment [18]. In the current study, younger patients were more likely to express difficulty with the scheduling process than over 55s. This aligns with a US radiology survey where patients aged 20–29 were least satisfied with MRI services [25]. Furthermore, in the current survey, greater difficulty was experienced by those who called more than once and tried to get an earlier appointment and among those who called multiple hospitals in an attempt to schedule a reasonable appointment. In a survey conducted by the Ministry of Health among 15,000 patients in 2018, only 64% of respondents said that they were able to schedule an appointment the first time they called the MRI department [26]. In the same survey, 80% said they received the test results within the given timeframe.

Patients who waited longer for the HMO approval of the MRI reported greater overall difficulty of the scheduling process. In a patient satisfaction survey from 2021/2, 95% of patients reported receiving reimbursement for tests they had requested, though 13% expressed difficulty in obtaining the approval/reimbursement [27]. It was also found that those that expressed difficulty were more likely to have chronic illness or language barrier [27].

Since the beginning of the October 2023 war, HMO agreements have been somewhat expanded with HMOs allowing their members to access tests in a greater number of places, in particular to accommodate those who have been evacuated from their home towns. Decision-makers may consider changing existing policy, which currently limits patients’ choices in access to healthcare, and makes the process more difficult; or consider adding a national call center to facilitate scheduling MRI appointments, showing all HMO facilities.

Currently we are in the process of implementing a national, retrospective measurement of WT for MRI appointments as well as for radiological reports. Integration of diverse data sources and perspectives—including WT by provider ownership and location, exam type, patient sociodemographics, personnel resources, infrastructure and insurer agreements—will help identify disparities between sub-populations and sites, and might inform interventions aimed at overcoming specific barriers and bottlenecks. New MRI scanners are being added to address long wait times, and accurate data can help identify where the need is greatest.

### Study limitations

Several limitations of the study must be taken into account. The survey was carried out via an internet panel and was sampled in order to get a representation of all the HMOs and all geographic regions in Israel. There was a certain underrepresentation of the Arab population, of HMO number 4, and of the southern district and Jerusalem, slightly below their representation in the population. While we present demographic information of responders vs non-responders, we do not have data on how many of the non-responders would have been relevant (i.e. had undergone an MRI in the past year in the public sector) yet chose not to complete the survey.

The waiting time estimate was approximate, measuring not the exact number of days but categories of time—we assumed that most participants would not be able to remember the exact number of days they waited, but would be able to estimate on a scale of weeks and months. Surveys are subjective and may entail recall bias, however they provide information on patients’ perception of WT which is important. The survey also did not include information on the clinical indication for an MRI test, only the type of test performed.

## Conclusion

According to a representative survey, many patients experienced some difficulty scheduling an MRI and almost two thirds waited over a month for an appointment. While this data was collected in Israel, it will be relevant to many health systems around the world where WT are prolonged. Alongside hospital metrics on WT, it is important to consider patients’ perspectives in scheduling imaging tests. In addition to attempts to reduce waiting times by adding scanners in the areas with the longest waits, and adding much-needed personnel, changes could be considered to streamline the appointment-scheduling process, make MRI WTs more transparent at different institutions, and to reduce the challenges patients face.

## Data Availability

Data can be provided on reasonable request.
